# Colocalized Sensing and Intelligent Computing in Micro-Sensors

**DOI:** 10.3390/s20216346

**Published:** 2020-11-06

**Authors:** Mohammad H Hasan, Ali Al-Ramini, Eihab Abdel-Rahman, Roozbeh Jafari, Fadi Alsaleem

**Affiliations:** 1 Mechanical and Materials Department, University of Nebraska–Lincoln, Lincoln, NE 68588, USA; mohammadhhasan@huskers.unl.edu (M.H.H.); aalramini@huskers.unl.edu (A.A.-R.); 2 Systems Design Engineering Department, University of Waterloo, Waterloo, ON N2L 3G1, Canada; eihab@uwaterloo.ca; 3 Electrical and Computer Engineering Department, Texas A&M University, College Station, TX 77843, USA; rjafari@tamu.edu; 4 Durham School of Architectural Engineering and Construction, University of Nebraska–Lincoln, Omaha, NE 68182, USA

**Keywords:** MEMS, reservoir computing, colocalized sensing and computing, neuromorphic computing, MEMS accelerometer

## Abstract

This work presents an approach to delay-based reservoir computing (RC) at the sensor level without input modulation. It employs a time-multiplexed bias to maintain transience while utilizing either an electrical signal or an environmental signal (such as acceleration) as an unmodulated input signal. The proposed approach enables RC carried out by sufficiently nonlinear sensory elements, as we demonstrate using a single electrostatically actuated microelectromechanical system (MEMS) device. The MEMS sensor can perform colocalized sensing and computing with fewer electronics than traditional RC elements at the RC input (such as analog-to-digital and digital-to-analog converters). The performance of the MEMS RC is evaluated experimentally using a simple classification task, in which the MEMS device differentiates between the profiles of two signal waveforms. The signal waveforms are chosen to be either electrical waveforms or acceleration waveforms. The classification accuracy of the presented MEMS RC scheme is found to be over 99%. Furthermore, the scheme is found to enable flexible virtual node probing rates, allowing for up to 4× slower probing rates, which relaxes the requirements on the system for reservoir signal sampling. Finally, our experiments show a noise-resistance capability for our MEMS RC scheme.

## 1. Introduction 

### 1.1. Motivation

Advances in complementary metal-oxide-semiconductor (CMOS) technologies resulted in an increasing reduction in the size and cost of transistors and sensory elements. As a result, great effort is being put towards incorporating sensors and microprocessors into various devices to create ‘Smart Devices’. These devices size range from large (smart cars [1,2], smart homes [3,4,5]) to minuscule (smart watches [6,7] and other wearable devices [8,9,10]). Consequently, data are being generated and collected at an ever-growing rate, with projections of continual growth. Emerging CMOS technologies have also enabled the rise of advanced robotic technologies, such as soft-robots that utilize new flexible electronic technologies, and micro-robots, which were realized by microelectromechanical systems (MEMS) sensors and fabrication techniques.

Processing data in smart devices, however, has proven to be a struggle. For example, smaller smart systems possess limited size and power capacities, which limit them to only perform simple computation locally or transmit their data to be processed in the cloud. However, improvements in computing power are plateauing as we approach the end of Moore’s law due to reaching the physical limits of transistor sizes miniaturization [11] and the complicated thermal management. Therefore, there is a great need for new computing architecture suited to the challenges of smart systems. These computing architectures must:

1. Take advantage of the large amount of data generated by the sensors without overwhelming the digital processors in the systems, i.e., enabling modularity.
2. Be power- and size-efficient, cutting some sources of energy loss in this computing architecture, such as analog-to-digital converters (ADC) and memory buses.

Ideally, to avoid latencies and wireless communication costs, the proposed computing architecture should be as close to the sensory node as possible to improve performance, as shown by the potential of edge computing architectures [12]. In this paper, an analog-based colocalized sensing-and-computing architecture is presented using MEMS sensors. Computing is performed immediately at the sensor node to compress analog data into meaningful information (e.g., in an activity monitoring wearable device, acceleration data would be sent as the number of footsteps taken), which enables the production of modular, intelligent, specialized sensors that are well suited for use in intelligent systems. Here, we rely on the concept of reservoir computing (RC) as a novel computing scheme that can be incorporated using sufficiently complex nonlinear analog elements while being well suited for time-series analysis.

### 1.2. Literature Review

Analog computing was popularized with the introduction of neuromorphic computing by Mead [13]. Neuromorphic computing was initially defined as the use of analog circuits, namely transistors in the sub-threshold regime, rather than the typical digital operational regime, to model the dynamics of spiking neuronal networks in the human brain. The concept was later extended to include the use of digital elements and hybrid analog–digital circuits to perform the same goal [14].

Neuromorphic computing schemes have demonstrated impressive computational abilities while retaining a small footprint. A recent neuromorphic computing scheme named reservoir computing (RC), first introduced independently by Mass [15] and Jaeger [16] under the names Liquid State Machines (LSM) and Echo State Networks (ESN), respectively, stands at the forefront of non-conventional (neuromorphic) computing. In RC, large networks of nonlinear nodes with randomly chosen connection weights, named a reservoir, project input signals into a high-dimensional space to enable computation, as shown in random automata [17], quantum RCs [18,19], and arrays of spin-torque oscillators [20]. The large nonlinearity induced by the network, coupled with a forgetting echo-state property enables mapping the reservoir output into a desirable output using simple linear regression, according to the Stone–Weierstrass theorem [21]. Recently, single delay-based dynamical systems were shown to express the same dynamics as RC networks of many neuronal nodes by virtue of their ‘infinite dimensionality’ [22,23]. In such systems, the reservoir is composed of the states of the dynamical system at fixed time intervals. Information is retained within this system by maintaining transience in the system through input time multiplexing [22]. Delay-based RC has been demonstrated using Boolean nodes [24], photonics [25,26,27,28], spin-torque oscillators [29], coupled oscillators [30], magnet arrays [31], and recently, MEMS devices [32,33]. Among these physical devices, photonics are the most popular due to their extremely high processing speeds. However, these devices require very long fiber optic wires to incorporate delayed feedback. They also require dedicated circuits for analog-to-digital and digital-to-analog conversion, signal processing, and sampling and holding, which increases the cost of the RC system. Furthermore, the high processing speeds of photonic RC systems necessitate the use of expensive sampling elements that may be infeasible for practical implementation.

In contrast, MEMS devices can operate in the range of hundreds of Hz to MHz, allowing higher timescale and sampling flexibility in the RC scheme. Furthermore, MEMS devices are attractive for embedding a new class of delay-based RC as they can potentially perform sensing and computing co-locally, which bypasses the sensing/computing layers separation complexity inherent in traditional RC. However, performing delay-based RC using sensory elements conflicts with the requirements of input time multiplexing, as sensed input signals are typically physical quantities like acceleration which cannot be multiplexed. 

### 1.3. Contribution and Paper Organization

In this work, we demonstrate the advantages of enabling a MEMS device to perform simultaneous sensing and computing by decoupling the reservoir inputs into physical sensory inputs and a bias time-multiplexing signal, which preserves the operation of the MEMS RC. Furthermore, we study the operation of the developed MEMS RC in a noisy environment by performing a non-trivial classification task. In particular, the contributions of this work are as follows:

1. This paper presents an RC scheme using a sensory element showing a demonstration of colocalized sensing and computing, enabling full edge computing.
2. This paper modifies the RC scheme to reduce the need for digital components. Specifically, this paper presents an approach to eliminate the need for input time multiplexing, thus reducing the need for a digital signal processor to produce the reservoir input.
3. This paper demonstrates further ability to reduce the computational costs of analog RC systems by reducing the virtual node probing rates, thus requiring less expensive sampling circuits at the reservoir output.
4. This paper experimentally tests the use of MEMS RCs in a noisy environment.

The structure of this paper is as follows: In Section 2, we describe the theory of reservoir computing, the dynamics of MEMS, and our approach of incorporating RC into the MEMS dynamics. Moreover, we describe the experimental setup used in this work in this section. In Section 3, we present our results, validating the use of the modified time-multiplexing scheme by performing a classification task using our MEMS RC. We then perform simultaneous sensing and computing using our MEMS RC scheme. Finally, in Section 4, we discuss our results and provide our conclusion.

## 2. Materials and Methods 

### 2.1. Reservoir Computing

**Figure 1:**
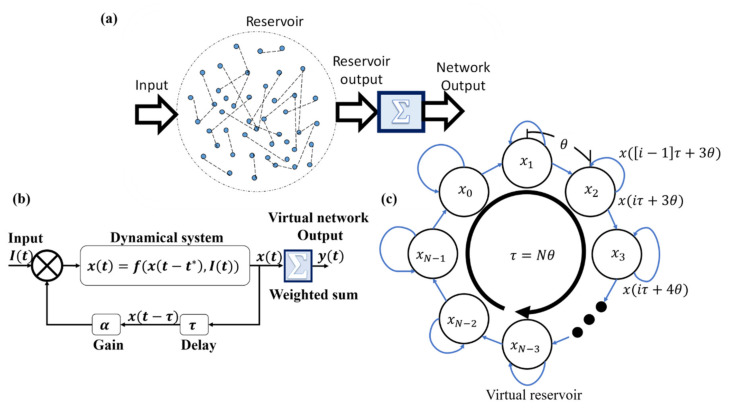
Reservoir computing (RC) schemes. (**a**) Classical RC utilizes a large network of randomly connected neurons. The output of the reservoir is a simple weighted sum of the neuronal response. (**b**) Delay-based RC utilizes the properties of dynamical systems and delayed feedback. (**c**) The virtual reservoir produced from delayed feedback and system dynamics.

Traditional reservoir computing architectures are composed of an input signal, a reservoir of nonlinear nodes with random connection weights, and some tunable output interface, as shown in Figure 1a. When the eigenvalues of the random connection weights are below unity, the interaction between the nodes retains complexity while remaining bounded. Therefore, the input of the reservoir is projected into a higher dimensional space, enabling computation [15,16].

Delay-based RC replaces the physical reservoir by a dynamical system coupled within a delayed feedback loop, as shown in Figure 1b. In this approach, instead of physical nodes, the dynamical system represents a network of N coupled virtual nonlinear nodes in time, at which the state of the dynamical system at a specific time step θ represents a virtual node. A virtual node is coupled to its preceding nodes due to the dynamical system’s dependency on previous time states. Self-coupling is also enabled through delayed feedback of a gain α and a time delay τ=Nθ [22], which produces a lower triangular adjacency matrix in the system [23], as shown in Figure 1c. 

The RC response of the system can be represented using a states matrix notion X, as follows:

(1) $$X_{i,j}=x_{j}(i)=x((i-1)\tau +j\theta ),i=1,\ldots ,T,j=0,\ldots ,N-1$$

where the dynamical system state $x\left(t\right)=f\left(x\left(t-t^{*}\right),I\left(t\right)\right)$, $f\left(.\right)$ is some update function for the dynamical system state, t is time, $t^{*}$ is some past time value(s) and $I\left(t\right)$ is an external input term. 

High coupling between the virtual nodes is achieved by retaining the dynamical system transience [28] through input time multiplexing [22]. Time multiplexing is usually performed using a sequence of analog-to-digital conversion, sampling-and-holding (for the duration of τ), and masking. Therefore, the reservoir input signal is given by Equation (2):

(2) $$J^{*}(t)=w_{j}\times I((i-1)\tau ),(i-1)\tau +j\theta \leq t<(i-1)\tau +(j+1)\theta ,i=1,\ldots ,T,j=0,\ldots ,N-1$$

where $J^{*}\left(t\right)$ is the input time-multiplex signal for the reservoir, $w_{j}$ is a periodic mask with period τ, usually chosen to be binary. The signal is a segmented piecewise constant function with a segment length of θ. $I\left(t\right)$ is the reservoir input at time t. Here, the input signal is assumed to be electrical to enable pre-processing through electrical time multiplexing. This approach, however, is ill suited for use in applications in which the input is supplied to the reservoir directly as a non-electrical signal. For instance, a MEMS accelerometer used as a reservoir cannot modulate the input acceleration waveforms it senses, as the signal measured within the MEMS device itself is non-electrical. However, the MEMS accelerometer may be used as a reservoir by using an electrically time-multiplexed accelerometer signal from another measurement device. In this case, sensing and computing are disjointed due to the need for electrical input time multiplexing. 

The output of RC is calculated by utilizing an output weight matrix $W_{o}$ to generate the output $Y^{*}$ that is updated at intervals of τ when all virtual nodes are processed using a weighted linear summation Equation (3):

(3) $$Y^{*}=X\times W_{0}$$

where $Y^{*}$ is the RC output vector: $Y^{*}={\left[y_{1}^{*},\;y_{2}^{*},\;\ldots ,\;y_{T}^{*}\;\right]}^{T}$, $y_{i}^{*}=y^{*}\left(i\tau \right),\;i=1,\ldots ,T$.

The weight vector can be simply trained using linear regression, or other simple regression methods, such as Ridge regression [34].

### 2.2. Micro-Electro-Mechanical-System Dynamics

**Figure 2:**
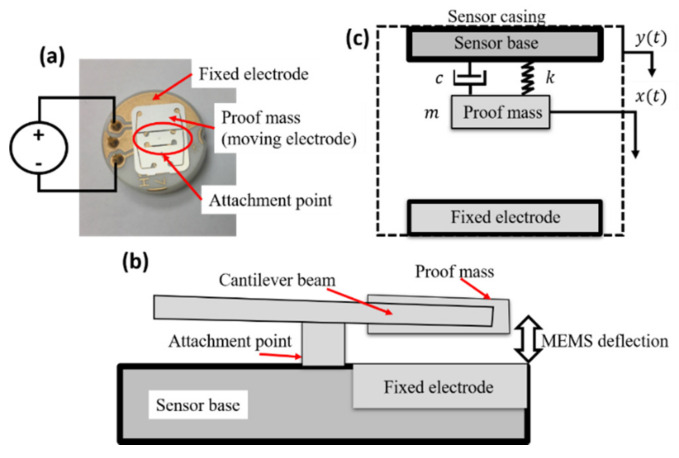
Microelectromechanical systems (MEMS) device schematics. (**a**) MEMS accelerometer used in this study. (**b**) Schematics of the MEMS side view showing the fixture the electrostatic gap. (**c**) Single-degree of freedom model of the MEMS accelerometer. Here, $c=2\zeta m_{eff}\left(2\pi f_{n}\;\right)$ is the damping constant and $k={\left(2\pi f_{n}\right)}^{2}m_{eff}$ is the linear stiffness of the MEMS device.

**Figure 3:**
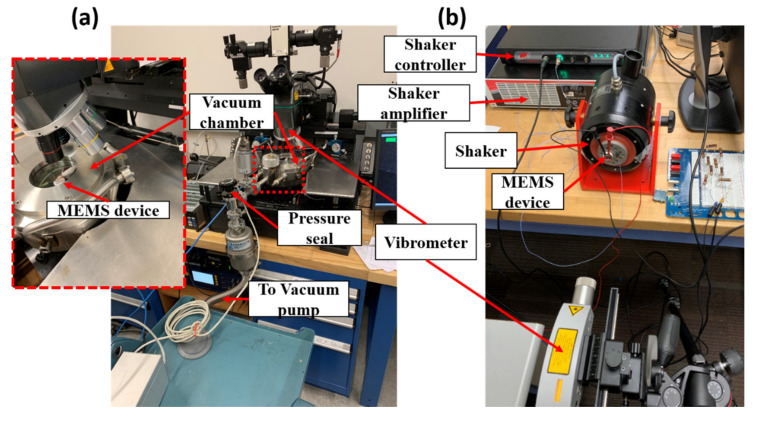
(**a**) Experimental setup for electrical waveform classification. The MEMS device is placed in a vacuum chamber to reduce pressure. The MEMS device serves as the reservoir in the RC system. The readout circuit is incorporated using an external data digital processor. The deflection of the MEMS device is attained by integrating the velocity signal from the laser vibrometer. (**b**) Experimental setup for acceleration waveform classification. The MEMS device is fixed on a shaker. The MEMS response is measured as the difference between the microbeam and ceramic base deflections. The shaker is controlled through a dedicated adaptive controller.

A doubly-cantilever, electrostatically-actuated MEMS accelerometer shown in Figure 2a,b is considered for our study. The MEMS device is considered the core of our reservoir computing scheme. The response of the MEMS accelerometer is simplified as a single-degree of freedom (SDF) spring-mass-damper system as shown in Figure 2c with dynamics governed by [35]:

(4) $$\ddot{z}(t)+4\pi f_{n}\zeta \dot{z}(t)+{\left(2\pi f_{n}\right)}^{2}z(t)=\frac{\varepsilon AV_{MEMS}^{2}}{2m_{eff}{\left(d-z\right)}^{2}}-\ddot{y}(t)$$

where *z(t) = x(t) − y(t)* is the relative displacement of the MEMS microbeam *x(t)* with respect to the motion of the base *y(t)* displacement at time t, f_n is the natural frequency, *ζ* is the damping ratio, ε is the electrical permittivity, A is the MEMS surface area, V_MEMS is the electrical voltage signal applied to the MEMS device and is usually made of a bias constant voltage V_b imposed with an AC voltage V_AC, m_eff is the effective MEMS mass, d is the nominal separation distance between the MEMS electrodes, and the dot operators represent temporal derivatives. The parameters of the used MEMS device are given in Table 1. While the dimensions are relatively large, the MEMS still exhibit the same dynamics as smaller MEMS devices due to the micro-scale d, as demonstrated in [18]. Additional information about parameter extraction and the frequency response of the MEMS accelerometer used in this paper is also available in [36]. The virtual nodes of the MEMS reservoir are represented by the state of the MEMS device *z(t)*, attained using the experimental apparatus in Figure 3.

### 2.3. MEMS Reservoir Computing

While the traditional approach of input time multiplexing in Equation (2) can work for the conventional MEMS-based reservoir (pure computing) by modulating the input AC voltage signal [32], this approach, however, is inappropriate for our novel concept of simultaneous sensing and computing. In this new concept, the input to the device will be non-electrical in nature (acceleration, force, etc.) that cannot be modulated. To overcome this challenge, we propose to time multiplex the bias voltage as follows:

(5) $$J(t)=w_{j}\times V_{b},(i-1)\tau +j\theta \leq t<(i-1)\tau +(j+1)\theta ,i=1,\ldots ,T,j=0,\ldots ,N-1$$

Similarly to a traditional bias signal, the proposed bias time-multiplexed signal is supplied to ensure the response MEMS remains transient to facilitate node coupling, which occurs when$\;\theta <1/\left(2\pi \zeta f_{n}\right)$. Using bias time multiplexing improves the performance of the RC by eliminating the need for sample-and-hold circuits and analog-to-digital conversion, which are otherwise necessary for input multiplexing in traditional delay-based RC. Thus, reducing the size and power consumption of the RC unit. Moreover, due to the elimination of the sample-and-hold circuit, the MEMS RC is capable of reacting to input signals that are faster than the sampling time, τ. Traditional RCs may address this limitation by increasing the sampling frequency beyond 1/τ. However, increasing the sampling rate further increases the RC power requirement.

The response of the virtual nodes in the MEMS reservoir is probed by measuring the relative MEMS deflection $z\left(t\right)$ at θ intervals to construct the virtual states X matrix in Equation (1) for the RC scheme. Ideally, probing should be done slightly after the injection of the time-multiplexing signal with a small delay of δ to enable the MEMS device to respond to the new input. This is performed experimentally by measuring the MEMS response by a temporal amount δ, with δ<θ. The ideal δ chosen is determined by trial and error allowing the maximization of the RC performance.

In the next sections, we show the performance of the MEMS RC using the modified RC scheme of using for computing only. Afterward, we show the performance of the MEMS RC in a sensing-and-computing task. The computational task chosen is a binary classification signal, in which the MEMS sensor is used to distinguish between two input waveforms: rectangular waveform and triangular waveform. This task, while simple, is non-trivial [37,38]. Furthermore, it enables simple replication, especially in the sensing-and-computing task due to the ability to generate similar signals using a vibration shaker. 

### 2.4. Experimental Procedure

The velocity response of the MEMS device is measured using laser vibrometers as shown in Figure 3a,b. The MEMS device is tested under both atmospheric pressure and reduced pressure (in a vacuum chamber) to study the effects of damping on our RC. The MEMS device is either excited using electrical signal [32] or mechanical acceleration signals $\ddot{y}\left(t\right)$. The electrical signal is fed as voltage values produced by a data acquisition device while the mechanical signal is generated using a vibration shaker as shown in Figure 3b. In both cases, the MEMS are supplied by a biasing voltage of 3 V and amplified by a 20 dB amplifier. This biasing voltage is used to produce the bias time-multiplexing signal using a binary random mask $w_{j}=\left\{0.3,1\right\}$ with a 90% chance of the mask taking the value of 1. When an electrical signal classification test was performed, an additional voltage signal was also applied prior to the amplifier, representing random clumps of rectangular and triangular signals with an amplitude of 3 V. This signal is removed in the mechanical signal classification test. The mechanical acceleration inputs are applied to the MEMS device using a vibration shaker. The shaker is controlled using a dedicated adaptive controller to produce signals very close to ideal rectangular and triangular signals.

The RC reservoir parameters chosen in this work are N=100, θ=1 ms and τ=100 ms. These parameters are chosen to ensure transience is maintained while decoupling the virtual nodes from the i timestep from nodes at the i+1 timestep [9], while maintaining coupling between virtual nodes within the ith computing period. For tasks with low memory requirements, such as classification, recurrent connections of the virtual nodes induced by delay-feedback may be forgone with limited impact on RC performance [39]. Therefore, α = 0 is chosen henceforth.

Acquiring the relative MEMS deflection $z\left(t\right)$ is performed by integrating $\dot{z}\left(t\right)=\dot{x}\left(t\right)-\dot{y}\left(t\right)$ after performing a high-pass filter to eliminate the low-frequency drifting often exhibited in accelerometers, where $\dot{x}\left(t\right)$ is the MEMS microbeam velocity measured at the moving electrode using a laser vibrometer and $\dot{y}\left(t\right)$ is the shaker base velocity measured by integrating an accelerometer signal. Both $\dot{x}\left(t\right)$ and $\dot{y}\left(t\right)$ are measured simultaneously and synchronized automatically using the vibration shaker controller. We note here that measuring $\dot{y}\left(t\right)$ is unnecessary when the MEMS device is actuated electrically as $\dot{z}\left(t\right)=\dot{x}\left(t\right)$ in this case.

The measured $z\left(t\right)$ is later down-sampled to a frequency of 1/θ to construct the X matrix following Equation (1). For binary classification, the RC is connected to two independent classifiers (readout circuits), one for each signal type. The expected outputs of the RC scheme are generated at intervals of τ as follows:

(6) $$Y_{R}=\left\{\begin{array}{l} 1,\;\mathrm{Rectangular}\;\mathrm{input}\\ -1,\;\mathrm{Triangular}\;\mathrm{input} \end{array}\right.$$

(7) $$Y_{T}=\left\{\begin{array}{l} -1,\;\mathrm{Rectangular}\;\mathrm{input}\\ 1,\;\mathrm{Triangular}\;\mathrm{input} \end{array}\right.$$

where $Y_{R}$ and $Y_{T}$ are the expected outputs of the rectangle classifier and triangle classifier, respectively. The matrix X and the vectors $Y_{R}=X\times W_{oR}$ and $Y_{T}=X\times W_{oT}$ are split into a training set and a testing set using an 80%–20% split. The training set is used to train the actual output of the rectangle and triangle classifiers, $Y_{R}^{*}\;$and $Y_{T}^{*}$, by using Ridge regression [34] to optimize the output weight matrices $W_{oR}$ and $W_{oT}$, respectively. Finally, the MEMS RC classification output is determined using a winner-take-all (WTA) scheme; defining success as the output of the classifier corresponding to the input waveform being higher than that of the other classifier. The success rate is calculated as:

(8) $$\mathrm{Success}\;\mathrm{Rate}=\frac{\text{\#}\mathrm{TestingSetCorrectClassifications}}{\text{\#}\mathrm{SamplesInTestingSet}}\times 100\%$$

The pseudocode that demonstrates the process of actuating and propping the MEMS RC response is presented in Algorithm 1 (Noting that $T_{s}$ is the sampling rate of velocity signals of the MEMS device and the shaker). Another pseudocode for postprocessing is presented in Algorithm 2.
**Algorithm 1** Interface with MEMS device1: Input *N*, *θ* 2: Input *V_b_*
3: Input *T_s_*= *θ/*100 4: Generate *w* = *rand*[*N,*1] 5: Perform thresholding on *w* to change to binary mask 6: **for**
*i* = 1*,*2*,...,T*
**do** 7: **for**
*j* = 1*,*2*,...,θ*
**do** 8: Generate and Maintain *J* = *w*[*j*]∗*V_b_*using data acquisition system 9: **for**
*k* = 1*,*2*,...,*100 **do** 10:     Acquire MEMS velocity $\dot{x}$ from vibrometer 11:     Acquire shaker velocity $\dot{y}$ from shaker controller  12:    Store $\dot{x}$ into array $\dot{x}$***Array*** 13:    Store $\dot{y}$ into array $\dot{y}$***Array*** 14:    Wait ***T_s_*** 15:   **end for** 16:  **end for** 17: **end for**

**Algorithm 2** Postprocessing1: Compute MEMS relative velocity array $\dot{z}$*Array* = $\dot{x}$*Array* − $\dot{y}$*Array* 2: Generate Expected Output array of rectangle classifier *Y_R_*of length *T* 3: Generate Expected Output array of triangle classifier *Y_T_*of length *T* 4: Initialize *X* 5: Input *δ* 6: Apply low-pass filter to $\dot{z}$*Array* 7: Compute *zArray* by integrating $\dot{z}$*Array* 8: Shift *zArray* by *δ/T_s_* elements 9: Downsample with sample rate *θ*: *zArray* → *zArrayDownSampled* 10: **for**
*i* = 1*,*2*,...,T*
**do** 11:     Fill X[i,ALL] = *i*th chunk of *N* elements of *zArrayDownSampled* 12: **end for** 13: Input *TrainSamples* 14: Generate *X_Train_* = *X*[1 :* TrainSamples,ALL*] 15: Generate *Y_R,Train_* = *Y_R_*[1 : *TrainSamples*] 16: Generate *Y_T,Train_* = *Y_T_* [1 :* TrainSamples*] 17: Generate *X_Test_* = *X*[*TrainSamples* + 1 : *T,ALL*] 18: Generate *Y_R,Test_* = *Y_R_*[*TrainSamples* + 1 : *T*] 19: Generate *Y_T,Test_* = * Y_T_* [*TrainSamples* + 1 : *T*] 20: Generate trained weight of rectangle classifier *W_oR_*= ${\left(\;XTrain^{T}\;XTrain\right)}^{-1}$∗ $\left(XTrain^{-1}\;YR,Train\right)$ 21: Generate test set results of rectangle classifier $Y_{R}^{*}$ = *X_Test_W_oR_* 22: Generate trained weight of triangle classifier *W_oT_* = ${\left(\;XTrain^{T}\;XTrain\right)}^{-1}$∗ $\left(XTrain^{-1}\;YT,Train\right)$ 23: Generate test set results $Y_{T}^{*}$ = *X_Test_W_oT_* 24: Compute classification accuracy *Success Rat*

## 3. Results

Two tests are considered: a test for MEMS as a simple computing unit and a test for MEMS colocalized sensing and computing. In both tests, we used the new concept of bias time multiplexing. Moreover, the classification tasks are performed in various pressure conditions to study the influence of damping on the performance of the RC scheme. Finally, the effect of noise is considered in these tests.

### 3.1. MEMS Reservoir Computing

We first test the MEMS in the worst-case scenario of operation at atmospheric pressure, resulting in high damping due to the squeeze damping effect. Operating at atmospheric pressure results in rapidly decaying transients, which may result in the development of a virtual reservoir with limited connectivity and low memory retention. The input to the MEMS device is a train of rectangle and triangle electrical signals with an amplitude equal to the bias voltage. The input voltage waveform is a random mix of periods of constant length of square and triangle signals with the same frequency and amplitude. To evaluate the response of the RC as a function of the input signal frequency, the input signal frequency is varied from 0.37%$f_{n}$ to 102%$f_{n}$ with a duty cycle of 50%. At each input frequency, the measured MEMS response is used to construct the virtual states matrix X by down-sampling the measured signal at θ = 1 ms. 

**Figure 4:**
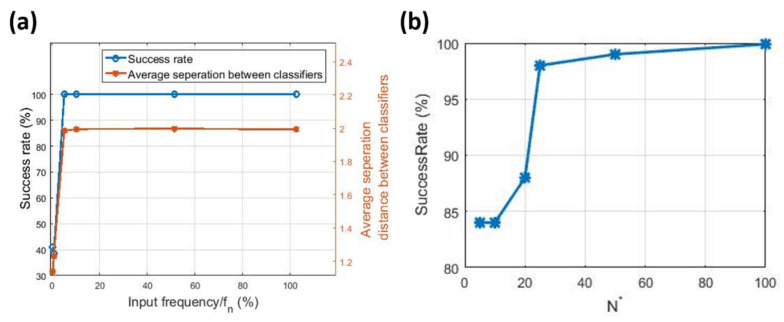
(**a**) Classifier accuracy as a function of the input frequency. At low input frequencies, the classifier fails to classify the input signal. However, as the frequency increases, the reservoir prediction accuracy increases to >99%. (**b**) MEMS RC classification performance at $f/f_{n}\;$ = 20% when different N are considered.

We find that while operating the MEMS at atmospheric pressure increases squeeze film damping and may produce a shallow reservoir with sparsely connected nodes, it successfully classified input signals with relatively high frequency, as shown in Figure 4a. Low input frequencies produce a quasi-static response, which is unsuitable for the reservoir computing scheme. 

The performance of the RC scheme can be directly inferred from the success rate. An alternative measure of performance is the average separation distance between the reservoir outputs $\bar{\left(X\times W_{oT}-X\times W_{oS}\right)}$, where the bar operator represents averaging, shown as the brown line in Figure 4a. A higher average separation distance signifies a more ‘confident’ classification by the RC. We also test modifying the number of nodes read $N^{*}$ while varying θ to maintain the same duration of computing (τ value). Figure 4b shows that $N^{*}=25$ produces a 98% success rate while reducing the required sampling rate in the system by 75% (θ=4 ms). This may indicate that some of the virtual nodes obtained at a higher sampling rate (θ=1 ms) might be redundant.

**Figure 5:**
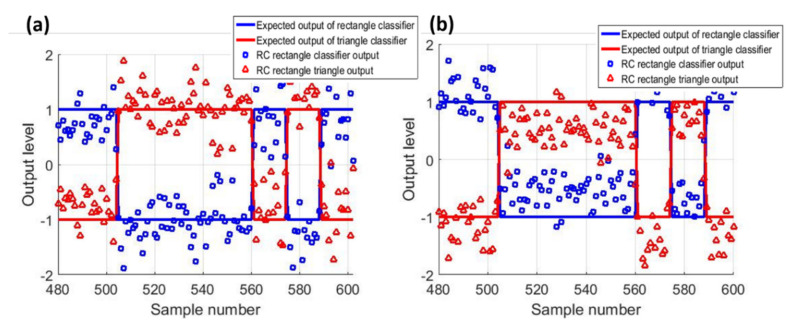
Experimental classification of low-frequency signal using the MEMS RC (**a**) under parameter (pressure) drift (**b**) and colored noise. The real-time results of the RC show success rates of 99.8% and 99.66%, respectively.

The frequency-dependent success in the presented results limits the utilization of the MEMS RC to tasks with relatively high-frequency input. To extend the operating frequency to quasi-static inputs, the memory of the system needs to be improved by reducing the squeeze film damping. This is achieved by reducing the operating pressure. Towards this end, the MEMS RC is placed in a vacuum chamber as shown in Figure 3a and is driven by an electrical signal at 0.37%$f_{n}$. Two types of variability are introduced separately to investigate the RC performance in classifying low-frequency signals in the presence of noise: (1) colored noise due to a combination of low-frequency ambient noise and ground vibrations resulting from running the vacuum pump, and (2) parameter drift as pressure built up in the vacuum chamber after shutting off the vacuum pump ahead of the experiment. Despite quasi-static input and the introduction of noise, the MEMS RC is capable of performing successful classification (pressure variation noise (99.8%, Figure 5a) and colored noise (99.66%, Figure 5b)). Importantly, compared to Figure 4, Figure 5 shows that the classification success rate of the MEMS RC when operated under reduced pressure improves its operation at the low input frequency.

### 3.2. MEMS Reservoir Sensing and Computing Unit

**Figure 6:**
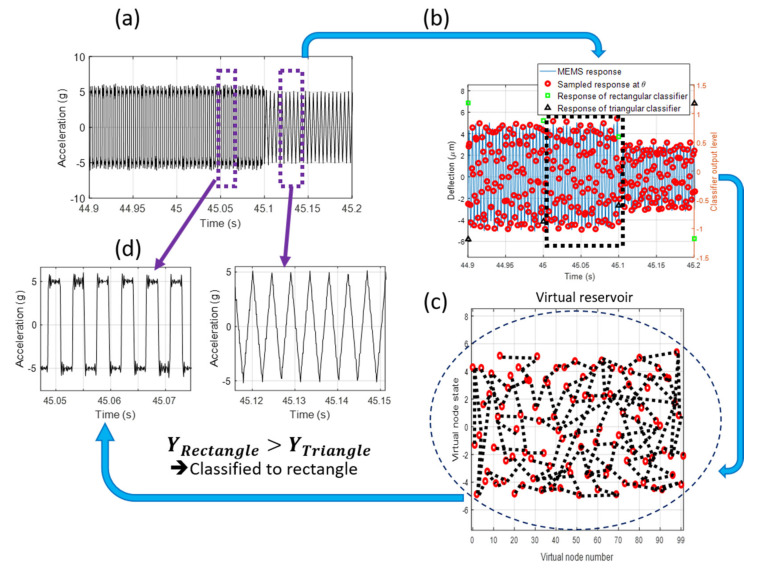
MEMS sense-and-compute scheme: (**a**) a sample of the acceleration signal generated by the shaker. (**b**) The response of the MEMS device and virtual node extraction. (**c**) Visualization of the virtual reservoir within the network. The response of the virtual nodes shown in this figure is used to compute the reservoir output after all the virtual nodes are updated. (**d**) Classification process based on the RC output.

The main advantage of using MEMS as an RC is the ability to perform sensing and computing simultaneously. In this case, MEMS can directly extract complex information from its environment. As an example, we present the first demonstration of a MEMS accelerometer that can directly classify a train of acceleration signals. The acceleration train is generated using a closed-loop controlled shaker, Figure 3b. The sensing and computing task is performed experimentally using an acceleration pulse train with a frequency of 1.1367$f_{n}$ and an amplitude of 5 g, Figure 6a. The frequency is the smallest frequency capable of producing sufficiently high acceleration to enable MEMS motion under the influence of squeeze-film damping at atmospheric pressure. The MEMS device is forced into transience using a time-multiplexed signal, which modulates the biasing voltage rather than the input signal. The relative displacement of the MEMS device is shown in Figure 6b. Figure 6c shows the MEMS RC response sampled and held each 1 ms, 80% of the data points are used to create the training set, and 20% of the data points are used for testing. After training, the MEMS network successfully classifies 99.6% of the testing data, which is on par with similar RC schemes for this test [31,37,38] and on par with the results observed from Figure 4. The success of this scheme shows that the sensing and computing using RC is possible using a slight modification to the input time-multiplexing scheme. We note here that the similarity between the results from the MEMS computing and the MEMS sense-and-compute tasks suggests that operation in a vacuum may still be required in colocalized sensing and computing tasks for low-frequency acceleration signals.

## 4. Discussion and Conclusions

**Figure 7:**
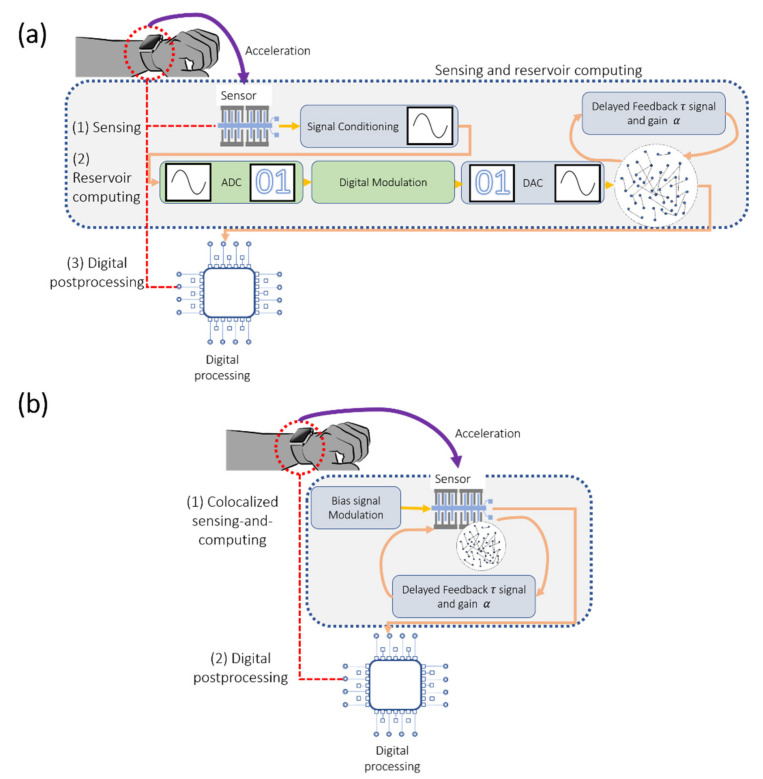
Illustration demonstrating the size reduction of the RC scheme with the use of sensors for colocalized sensing-and-computing. In this figure, digital components are represented by green blocks. Analog components are represented by grey components. (**a**) Delay RC scheme with separated sensing and computing. (**b**) Delay RC scheme with colocalized sensing and computing.

The use of input-based time multiplexing was previously shown to be beneficial for implementing RCs using a single physical dynamical node. However, this scheme requires injecting data into the reservoir by using a combination of external sensory elements and computationally inefficient analog-to-digital converters (ADCs) to enable input time multiplexing and digital-to-analog converters (DACs) to supply the input signal into the analog reservoir. These external components can be eliminated by using sensory elements, such as colocalized sensing-and-computing nodes, where information is simultaneously gathered and processed, as shown in Figure 7. This process can be achieved by utilizing a bias voltage signal for time multiplexing rather than the input signal, thus enabling the RC to immediately perform computing on environmental signals (acceleration in this work) without compromising computational performance. Because the bias signal is time-multiplexed, rather than the input signal, the analog-to-digital conversion and digital modulation may be eliminated and replaced by an analog input time-multiplexing circuit with a fixed output. Delay-feedback may also be performed using analog electronics using capacitive circuits. Furthermore, amplification can be performed passively by relying on the mechanical and electrical resonances in MEMS devices, as was demonstrated in previous works [40]. Thus, the pre-processing and processing steps of the reservoir computer can be performed entirely in an analog fashion, which will be the subject of our future work. 

This approach is also shown to be resilient to different types of noise and successful even under high damping conditions. Moreover, it enables reducing the virtual node-probing period from θ to as high as 4θ, which can also improve the efficiency of the scheme by reducing the reservoir sampling rate.

The utilization of MEMS devices in the modified RC scheme enables sensing and computing. Additional benefits are expected when simultaneously targeting and exciting multiple mode shapes of a MEMS microstructure. In this case, the MEMS device is modeled as a contentious beam rather than a simple SDF model, which may enable the production of multiple independent virtual reservoirs with different timescales within the same MEMS device. Such a concept of using multiple reservoirs to boost the RC performance [41], especially asynchronous reservoirs [42], is recently shown to circumvent the performance plateau as the number of virtual nodes increases.

While previous works have shown the potential of performing simple edge computing using MEMS sensors [43], this approach shows a new generation of intelligent sensors can be produced without using large networks of sensory elements [44]. Such sensors will entirely utilize transience, increasing their speeds. Furthermore, these sensors may be capable of performing edge computation by performing classification and/or prediction. For example, RC sensors may be capable of compensating for interference due to measurement in nonlinear systems, such as compensation for flow changes due to the insertion of a flow rate sensor into a water pipe. 

In conclusion, in this work we demonstrated a reservoir computing scheme using a single MEMS sensor to perform colocalized sensing and computing to reduce the cost of RC implementation. The developed scheme modifies the approach used in traditional RC systems by eliminating the need for input time multiplexing, which reduces the need for digital electronics at the reservoir inputs to perform sample-and-hold, analog-to-digital conversion, digital processing and digital-to-analog conversion. Instead, the MEMS RC scheme utilizes a time-multiplexed bias signal (modulated bias) that may be generated using simpler components. The MEMS RC scheme was tested through solving a waveform classification problem, in which this waveform is provided either electrically (computing task) or as an acceleration signal (sensing and computing). In both cases, the MEMS RC yielded a classification accuracy > 99%, even in the presence of noise. Finally, this paper further reduces the cost of using RC by showing an acceptable (~98%) classification accuracy at reduced virtual node probing rates (up to 4x slower virtual node probing), reducing the required MEMS signal sampling rate. 

## Figures and Tables

**Table 1:** MEMS parameters.

Parameter	Physical Meaning	Value
ζ	Damping constant (in vacuum)	$3\times 10^{-3}$
$f_{n}$	MEMS natural frequency	195.3 Hz
ε	Electrical permittivity	$8.85\times 10^{-12}\;$F/m
A	MEMS surface area	$39.6{\;\mathrm{mm}}^{2}$
d	Nominal separation gap between moving and fixed electrodes	42 μm
$m_{eff}$	Effective MEMS mass	106 mg
